# Effect of Lactate Minimum Speed-Guided Training on the Fluid, Electrolyte and Acid-Base Status of Horses

**DOI:** 10.3390/ani13203290

**Published:** 2023-10-21

**Authors:** Angélica C. Titotto, Maíra M. Santos, Gabriel V. Ramos, Milena dos S. Adão, Guilherme V. Benvenuto, Luciana C. C. De Lacerda, Júlio A. N. Lisbôa, José C. Lacerda-Neto

**Affiliations:** 1 Department of Clinic and Veterinary Surgery, School of Agricultural and Veterinarian Sciences, São Paulo State University (Unesp), Jaboticabal 14884-900, SP, Brazil; angelica.titotto@unesp.br (A.C.T.); gabriel.ramos@unesp.br (G.V.R.);; 2 Department of Veterinary Clinics, State University of Londrina (UEL), Londrina 86057-970, PR, Brazil

**Keywords:** athletic horse, conditioning program, endurance, exercise physiology

## Abstract

**Simple Summary:**

Horses are susceptible to metabolic disturbances provoked by dehydration and electrolyte and acid-base imbalances that can affect their welfare and result in their elimination from competitions. The conditioning guided by physiologic variables such as lactate can bring greater gains in terms of conditioning, and therefore better body preparation for the exercise challenge. In this study, horses were submitted to a lactate minimum speed (LMS)-guided training program for six weeks, and its effect on their water, electrolyte, and acid-base statuses was measured. Even though it was found that this training method has a positive impact on the horses’ acid-base status during exercise, the protocol needs adaptation in order to maximize their fitness gain.

**Abstract:**

The effect of lactate minimum speed (LMS)-guided training on horses’ homeostasis is still unknown. Thus, this study aimed to evaluate the effect of an LMS-guided training program on the fluid, electrolyte and acid-base status of horses. Ten untrained Arabian horses were submitted to an LMS test on a treadmill before and after six weeks of training. The training intensity was 80% of the LMS in the first three sessions and 100% of the LMS in the other sessions. The venous blood was collected before (T-1) and after (T-2) training at rest, during and after the LMS test for lactate, pH, pCO_2_, HCO_3_^−^, and electrolyte measurements. The LMS and strong ion difference (SID_4_) were calculated. A mild increase in the mean values (*p* > 0.05) was observed at rest in T-2 in comparison with T-1 in the following variables: pH (from 7.436 ± 0.013 to 7.460 ± 0.012), pCO_2_ (from 42.95 ± 1.58 to 45.06 ± 0.81 mmHg), HCO_3_^−^ (from 27.01 ± 1.02 to 28.91 ± 0.86 mmol/L), and SID_4_ (from 33.42 ± 1.45 to 35.06 ± 2.94 mmol/L). During T-2, these variables were more stable than during T-1. Despite the improvement in fitness, the LMS did not indicate a significant difference (from 5.40 ± 0.55 to 5.52 ± 0.20 m/s). The results confirmed that the LMS-guided training program had a positive impact on the horses’ acid-base status, although some adaptations are still required to improve their fitness.

## 1. Introduction

Horses’ fitness is an important factor, especially in long-term sports such as those involving endurance in which metabolic disorders are the second cause of elimination from competitions. Metabolic problems associated with dehydration, electrolyte imbalance, heat accumulation and energy depletion can affect animal welfare [1]. In equines, fluid and electrolyte losses can lead to cardiovascular and thermoregulatory instability [2]. During exercise, respiratory activity, the movement of fluids and electrolytes, and changes in blood temperature can affect the acid-base status [3]. Since exercise and training modify the content of body fluids and electrolytes in horses [2], guided training can improve their conditioning and control homeostasis during exercise. 

Various parameters, e.g., maximum oxygen consumption ($\dot{V}$O2max), heart rate and blood lactate, can be used to obtain horses’ fitness [4]. Furthermore, some cardiac and muscular biomarkers such as cardiac troponin (cTnI), aspartate amino transferase (AST), alanine amino transferase (ALT), creatine phosphokinase (CPK), lactate dehydrogenase (LDH), and myoglobin have been widely used to assess their post-exercise response [5]. In this context, a complete blood count and serum biochemical can also be useful to evaluate the health status and performance of athletic equines [6].

Among these parameters, blood lactate accumulation is largely used to evaluate the fitness level and aerobic capacity of horses [7,8]. The velocity–lactate relationship allows determining the optimal training load so as to maximize their performance and prevent some sports injuries [7]. In addition, it is essential to evaluate their response after the training program in order to verify the occurrence of adequate musculoskeletal and cardiovascular adaptations. Although there are many standardized exercise tests, there is still a need for an easier and more reliable test. Additionally, to the best of our knowledge, the effect of lactate minimum speed-guided training on the fluid, electrolyte and acid-base status of horses has not been fully investigated yet.

Widely studied in humans, the lactate minimum speed (LMS) method is used for determining both their fitness and training [9,10]. Nonetheless, some aspects regarding the LMS test protocol, i.e., its predictive value of the maximal lactate-steady-state (MLSS), which represents the exercise intensity at which there is a balance between blood lactate production and removal, and effectiveness in the training of human athletes, still require further research [9]. Even though different methods using lactate have been applied in equine training programs [11,12], studies employing the LMS test in these animals are still scarce, and a few existing works have only used it to determine their fitness [13,14,15]. Recently, two studies employed an LMS-guided training protocol in horses to assess its effect on their blood osmolality [16] and conditioning, in addition to comparing its performance parameters with other effort tests [7].

**Figure 1:**
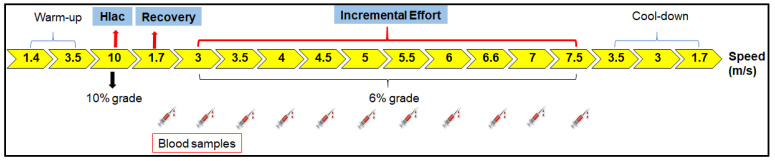
Effort test scheme. The lactate minimum speed (LMS) test performed on a treadmill consisted of three phases: hyperlactatemia (Hlac) induction, which is a phase of short, high-intensity exercise to induce hyperlactatemia; active recovery to provide time for lactate transposition from muscle cells to the bloodstream; and incremental effort. Blood samples were collected at the end of hyperlactatemia induction and during the effort test to measure lactate for LMS determination. The LMS test produces a “U-shaped” lactate curve. The same protocol test was applied before and after the training period. The test lasted 57 min, and the horses traveled about 800 m at each speed. The time spent at each speed was variable. More details can be found in the Section 2.

The LMS test was development by Tegtbur et al. [17] and consists of three phases: a phase of high intensity and short duration to induce hyperlactemia; a recovery phase to ensure time for lactate displacement from muscle cells to the bloodstream; and lastly, an incremental effort phase (Figure 1). This test produces a “U-shaped” lactate curve, as after hyperlactatemia, there is a predominance of blood lactate clearance, while during the incremental effort, the blood lactate concentration increases. The lowest point of this curve corresponds to the LMS, and therefore the balance between lactate clearance and production. The LMS test is an easy method to determine the lactate threshold and a good parameter to modulate the conditioning load for horses. It has a better correlation with the maximum lactate steady state than other standardized effort tests to evaluate the aerobic capacity of horses [7]. However, its use as a method to guide training programs for horses still needs to be further explored.

Therefore, this study aims to evaluate the impact of a six-week training program guided by lactate minimum speed determined in a treadmill effort test on the fluid, electrolyte and acid-base status of horses. We hypothesize that, after the conditioning program, these metabolic status factors will improve, mainly during exercise, confirming the usefulness of the LMS method in guiding horse training.

## 2. Materials and Methods

### 2.1. Animals

This research was approved by the Ethics Committee on the Use of Animals of the School of Agricultural and Veterinary Sciences (FCAV) of the São Paulo State University “Júlio de Mesquita Filho”, Jaboticabal, SP, Brazil (UNESP), under protocol no. 005128/18. The research included 10 clinically healthy adult purebred Arabian horses (geldings and mares) without lameness, with an average body weight of 380 ± 23.7 kg, without previous training and belonging to the FCAV/UNESP herd. The animals were kept in paddocks, with grass-based food (Tifton 85), water and mineral salt ad libitum. They were provided with a concentrated ration equivalent to 1% of body weight of each animal on a daily basis, according to the nutritional requirements for moderate work established for horses [18].

### 2.2. Study Design

Initially, the animals were submitted to a physical effort test (T-1) to establish the workload to be applied during the training. In this first test, the running speed corresponding to the minimum lactate concentration of each animal was determined. After the training period, the same effort test was repeated (T-2), and the results obtained were compared with the values observed in T-1. The effort tests (ETs) and training were performed at the Equine Sports Medicine Laboratory (LMEE) of FCAV/UNESP, which is equipped with a high-speed treadmill. The room temperature and relative humidity were maintained at 20 °C and approximately 50%, respectively. Prior to the beginning of the experimental phase, the animals underwent a treadmill adaptation period of three days and were then submitted to T-1.

### 2.3. Effort Test

The effort test (ET) consisted of warm-up, hyperlactatemia induction (HLac), incremental effort (IE) and active cool-down phases. The LMS test comprised the HLac phases, in which metabolic acidosis was induced, and the IE, which was performed in sequence (Figure 1). This protocol was defined based on previous endurance training experience since, when the study was carried out, there was no established LMS test protocol for horse training. The same ET was performed before the beginning (T-1) and after the six-week training period (T-2).

**Figure 2:**
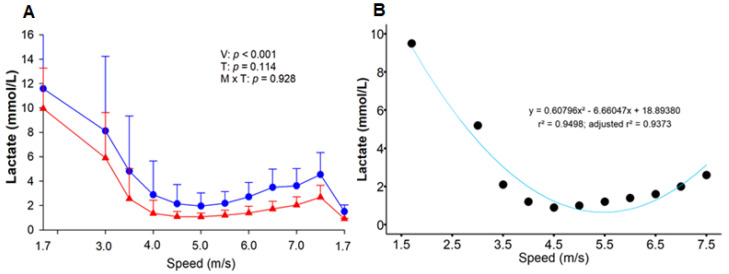
Effect of six weeks of training on the response of blood lactate accumulation during the lactate minimum speed test (**A**). Before (T-1; blue circle) and after (T-2; red triangle) the training period. V = velocity; T = effort test; V × T = moment × test interaction. Second-order polynomial function curve fitted to lactate concentration as a function of speed in a representative animal (**B**). The running speeds were: 1.7, 3.0, 3.5, 4.0, 4.5, 5.0, 5.5, 6.0, 6.5, 7.0, and 7.5 m/s. •, post-training lactate behavior.

Blood samples were collected at rest (M0) and at the end of certain stages of the ET to assess the acid-base status (Moment), as it was unnecessary to measure it at all moments. To evaluate the LMS method, the samples were collected from the end of HLac (1.7 m/s) to the end of the IE phases (7.5 m/s). The details of the ET can be found in Table 1. For each speed, the distance traveled by each horse was approximately 800 m. The LMS was calculated as the speed at which the lactate concentration reached its lowest value during the IET. To this end, second- and third-order polynomial function curves were fitted to the lactate curve. A first derivative of zero equivalent to the lowest point of the curve was assumed as the concentration of lactate corresponding to the LMS (Figure 2B) [19].

### 2.4. Blood Collection and Analysis

Prior to the ET, a catheter (14G) was attached to the horses’ left jugular vein, together with a 40 cm extension tube to facilitate blood collection with the animals on the treadmill. The system was filled with heparinized saline solution to maintain its viability, and before sample collection, the volume corresponding to this solution was discarded. To assess their acid-base status, blood samples were collected at the following moments: M0 (rest), M1 (end of HLac induction), M2, M3 and M4 (initial stages of IE), M5 (end of IE), M6 (end of active cool-down) and M7 (20 min after the end of ET). In addition to these moments, in order to evaluate the LMS, more samples were collected and analyzed during other periods of the test (from the Hlac induction phase at a speed of 1.7 m/s to the end of the incremental effort phase at a speed of 7.5 m/s), as displayed in Figure 1. The collections were performed at the end of each ET phase, with the treadmill turned off for 1 min. After the collection of the last sample, which was performed before the horses ingested water, the catheter was removed.

A volume of 4 mL of blood was obtained from each sample and placed in 4 mL tubes containing lithium heparin and separator gel (BD, Jundiaí, SP, BR), which were kept in a thermal box with water and ice. The samples were processed at LMEE, which also carries out laboratory analyses. The time between sample collection and analysis was 10 min. Measurements of blood pH, the partial pressure of carbon dioxide (pCO_2_), bicarbonate concentration (HCO_3_^−^), base excess (BE), concentrations of lactate, sodium (Na^+^), potassium (K^+^), chloride (Cl^−^) and ionized calcium (iCa^2+^), and packed cell volume (PCV) were carried out on a blood gas analyzer (Cobas b 123 Roche Ltda, MA, DE) at a standard temperature of 38 °C. The determination of total plasma protein (TPP) concentration was performed by refractometry (Atago Brasil Ltda, Ribeirão Preto, SP, BR) after plasma collection by centrifugation (1500× *g* for 10 min). The strong ion difference (SID_4_) [20], anion gap (AG), strong ion gap (SIG) [21], and total concentration of nonvolatile buffers (A_tot_) [20] were calculated (Table S1).

### 2.5. Training

Once the individual LMS was determined in T-1, the treadmill training period began. Each training session consisted of 5 min of warm-up (at 3.5 m/s), 40 min of training exercise, and 5 min of cool-down (at 3.5 m/s). The training intensity was increased every three sessions. In the first three sessions, the intensity established was 80% of the LMS and without any grade. Between the fourth and sixth sessions, the horses began to exercise at 100% of the LMS, with four periods of 5 min at a 2% grad interspersed with four periods of 5 min without any grade. From the seventh session onward, 100% of the LMS was used, alternating every four 5 min periods at an grade of 5% with four periods without any grade. The training was performed in an air-conditioned room and consisted of 5 sessions of 14 days each. The horses rested for one day between the sessions during the week. After six weeks of training, the horses performed a total of 15 exercise sessions of 5 min each. This program was designed based on previous endurance training experience. During the development of the research, there were no publications using the LMS method to guide horse training. After the training period, the horses were submitted to T-2 to determine the LMS.

### 2.6. Statistical Analysis

The data were analyzed using SigmaPlot for Windows (Systat Software Inc., v.13.1, San Jose, CA, USA) and are expressed as a mean ± SD. Two-way repeated measures analysis of variance (ANOVA) was used to test the effects of the effort test (before, during and after) the training (before and after) and the interaction between both. Tukey’s test was employed to compare the means when F-statistic was significant. Student’s *t*-test was used to compare the lactate minimum speed before and after training. Pearson’s correlation coefficient was applied to test relationships between the polynomial functions, while the limits of agreement were analyzed employing the Bland and Altman technique [22]. Values of *p* < 0.05 were considered statistically significant.

## 3. Results

The ten horses completed the conditioning program. Two horses presented lameness in the cool-down phase of T-2, thus requiring the interruption of the ET. However, the variables obtained from these animals until that moment were used in the analyses. A high correlation was obtained between the polynomial functions in T-1 (r = 0.899) and T-2 (r = 0.884) (*p* < 0.05). The limits of agreement for comparisons between the speed obtained by second- and third-order polynomial functions suggest a good agreement (Figure S1). The second-order polynomial function was used for adjustment. In T-1, the mean LMS value obtained was 5.40 ± 0.55 m/s, and the animals performed the three initial training sessions at 80% of this value, resulting in a mean speed of 4.3 m/s. In T-2, the LMS increased to 5.52 ± 0.20 m/s, but without any significant difference (*p* = 0.594). Throughout the IE phase, there was a tendency towards lower lactate concentrations in T-2, yet with no statistical difference (Figure 2A).

The lowest lactate values were observed at rest (M0), at the end of cool-down (M6) and 20 min after (M7), both in T-1 and T-2, with no difference between them (Table 2). The HLac phase significantly increased the lactate concentration (M1), causing it to reach its maximum value. Although the IE phase started with a high lactate concentration (M2), as the speed was increased, this variable gradually decreased until reaching its lowest value at M4, followed by a slight increase at M5.

**Figure 3:**
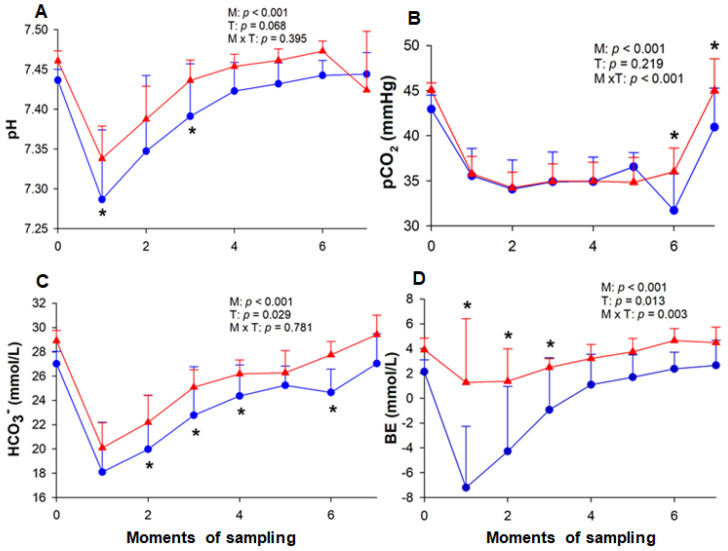
Variation of blood pH (**A**), pCO_2_ (**B**), HCO_3_^−^ (bicarbonate) (**C**) and BE (base excess) (**D**) in horses. The measurements were performed at rest (M0), at the end of hyperlactatemia (M1), at early stages (M2, M3 and M4) and the end (M5) of the incremental effort, at the end of cool-down (M6), and 20 min after the end of the test (M7). T-1 (blue circle) and T-2 (red triangle) correspond to before and after the training period, respectively. M = moment; T = effort test; M × T = moment × test interaction. * Significant difference between tests 1 and 2.

During both ETs, the pH decreased at the end of the HLac phase (M1) (T-1: 7.286 ± 0.087; T-2: 7.338 ± 0.040), remaining low at M2 (T-1: 7.347 ± 0.094; T-2: 7.387 ± 0.041) and returning to baseline values at M3 (T-1: 7.391 ± 0.065; T-2: 7.436 ± 0.025). The pH values differed between the tests at M1 and M3, being lower in T-1 (Figure 3). The exercise decreased pCO_2_ in both T-1 and T-2, returning to baseline at M7. In the last two collections, low values of pCO_2_ were observed in T-1 (M6: 31.71 ± 4.12 mmHg; M7: 40.95 ± 4.34 mmHg) when compared to T-2 (M6: 36.00 ± 2.64 mmHg; M7: 45.00 ± 3.52 mmHg). The HLac phase decreased the concentration of HCO_3_^−^ in both tests (M1) (T-1: 18.01 ± 4.11 mmol/L; T-2: 20.09 ± 2.06 mmol/L), returning to its baseline value at M7 in T-1 (27.07 ± 2.45 mmol/L) and M5 in T-2 (26.28 ± 1.82 mmol/L). Lower concentrations of HCO_3_^−^ were found in T-1. As for BE, there was a reduction in this value after the HLac phase (M1) in T-1 (−7.20 ± 4.93 mmol/L), which remained until M3. In contrast, no change was observed in this variable in T-2.

**Figure 4:**
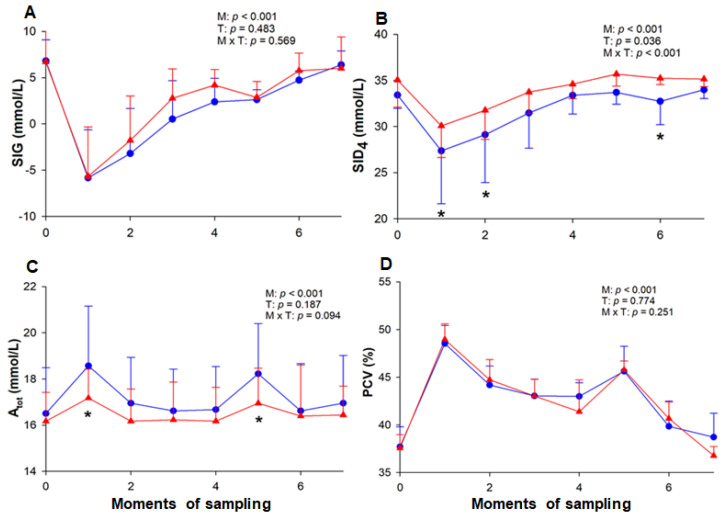
Variation of SIG (**A**), SID_4_ (**B**), A_tot_ (**C**), and PCV (**D**) in horses. A_tot_, total concentration of nonvolatile buffers; PCV, packed cell volume; SID_4_, strong ion difference; SIG, strong ion gap. The measurements were performed at rest (M0), at the end of hyperlactatemia (M1), at early stages (M2, M3 and M4) and the end (M5) of the incremental effort, at the end of cool-down (M6), and 20 min after the end of the test (M7). T-1 (blue circle) and T-2 (red triangle) correspond to before and after the training period, respectively. M = moment; T = effort test; M × T = moment × test interaction. * Significant difference between tests 1 and 2.

The baseline value of AG remained equal in both T-1 and T-2, followed by a significant increase in both ETs at the end of the HLac phase (M1) when it reached its maximum value (Table 2). In the beginning of the IE phase, AG was high in both T-1 and T-2 (yet below the value observed at M1), and as the speed was raised, this value gradually decreased until M4. At the end of the cool-down (M6), the AG values returned to baseline and were maintained. During the ETs, the SIG decreased at M1 and returned to its baseline value at M6 in T-1 and M7 in T-2. Between the ETs, the SIG values did not differ statistically (Figure 4). During T-1, there was an increase in A_tot_ and TPP (Table 2) at M1 and M5, whereas in T-2 these variables did not change. The PCV variation was the same in both ETs, increasing from M1 onward (T-1: 48.54 ± 1.89%; T-2: 48.86 ± 1.64%) until M7, when it returned to baseline (T-1: 38.71 ± 2.51%; T-2: 36.77 ± 0.94%) (Figure 4).

The concentrations of Na^+^ and Cl^−^ did not differ between the ETs (Table 3). Although there was an increment in Na^+^ at M1 in both ETs with a subsequent and gradual decrease, in T-1, the Na^+^ concentration increased again at M5. With respect to Cl^−^, there was a decrease in this value at the end of the exercise in T-2, which was reversed at M7, while at the same moment, in T-1, this concentration remained low. In both tests, the concentration of K^+^ increased during the exercise and returned to its baseline value at the end (M6). In contrast, it decreased in T-1 at M7 and reached higher values than those observed in T-2 at some moments. There was a gradual reduction in Ca^++^ in both ETs, and despite the slight increase recorded at M7, its concentration was still below the baseline value. A reduction in SID_4_ was observed at M1 (27.37 ± 5.75 mmol/L) and M2 (29.11 ± 5.19 mmol/L) during T-1, whereas, during T-2, it only decreased at M1 (30.07 ± 3.43 mmol/L) (Figure 4). When comparing both ETs, it could be noted that the SID_4_ presented lower values at some moments of T-1.

## 4. Discussion

The results confirmed that the six-week training program guided by the LMS test had a positive impact on the acid-base status of horses, according to the data obtained during T-2. Some researchers have used this method to determine horses’ fitness and concluded that it is useful and easy to be applied in the field [7,13,14,15]. To the best of our knowledge, this is the first study evaluating the effect of LMS-guided training on the fluid, electrolyte and acid-base parameters of horses. Even though there are controversies regarding the exercise protocol used, its effectiveness in monitoring the effect of training and its predictive value of maximal lactate-steady-state (MLSS) in humans [9], in equines, the LMS test is a valuable tool for assessing their aerobic capacity, as it shows a good correlation with MLSS [7].

The training employed herein improved the animals’ fitness, and although the lactate concentration did not differ between the ETs, there was a tendency towards a lower concentration during T-2, as well as an increase in LMS. It is reported that aerobic training improves both lactate clearance via monocarboxylic acid transports [23] and its use as energy by tissues not involved in the exercise, in addition to the fact that its production is lower [24]. Moreover, after the training period, the horses showed mild alkalemia at rest, with pH, pCO_2_ and HCO_3_^−^ values close to the upper limit considered physiological for the species (Table S2). This may be attributed to the enhanced buffering capacity induced by training previously reported in athletic horses [25,26], given that the animals were already adapted to the diet.

When applied in humans, the same exercise protocol before and after six weeks of endurance training showed that the LMS method was not sensitive enough to detect changes in their endurance capacity, which was confirmed by other tests [27]. Nevertheless, subsequent research proved the test sensitivity for evaluating the effect of training in humans [9]. It is known that the initial intensity of the IE phase after the training period must be adjusted due to improved fitness and faster lactate clearance [27,28]. This can explain why Carter et al. [27] did not find a change in LMS despite verifying a reduction in lactate concentration. Furthermore, the mathematical model used by the authors to determine LMS is questionable. Unlike the present work, they applied the spline function, which considers the nadir. Because of this, there may be some misinterpretations since lactate clearance tends to be faster due to fitness improvement, causing a left shift of the lactate concentration, as observed herein. To validate the LMS test in the longitudinal monitoring of training in humans, it is still necessary to evaluate whether the mathematical model influences the LMS determination, as there was an increment in speed in studies that applied the second order of polynomial function [9].

In contrast to the results found in the present study and in humans, De Maré et al. [7] noticed an improvement in the horses’ conditioning and a reduction in LMS after eight weeks of LMS-guided training. However, the training protocol consisted of interspersing aerobic and interval training. In addition to the greater training intensity used by the researchers compared with the present work, the training period may also have contributed to some differences in the results. In spite of being a valuable tool for evaluating the conditioning of horses without leading them to exhaustion, the LMS test is protocol-dependent, and thus requires some adaptations, mainly in the post-training [7].

In the present study, the horses showed metabolic acidosis with uncompensated respiratory alkalosis after the HLac phase in T-1, whereas in T-2, the acidosis was compensated. Although the acidemia observed in T-1 was reversed in the following moment, the blood pH tended to be lower in relation to T-2 throughout the test. Such negative correlation between lactate concentration and pH [29] can be attributed to the fact that the animals produced more lactate in T-1. Furthermore, there was a reduction in BE in T-1; however, this value did not change in T-2 despite the decreased concentration of bicarbonate in the initial phases of the effort test. As for AG and SIG, these values were higher in T-1. It is worth mentioning that these variables are reliable to predict the lactate concentration under conditions where the TPP concentration is within the reference limit or not, respectively [21]. In high-intensity exercise, the reduction in pH is related to an increase in the concentration of lactate and H^+^ ions as a result of the anaerobic metabolism of muscle cells associated with high energy demand, and in the case of H^+^ ions, muscle fatigue [30].

The fact that the compensatory response to lactate accumulation was more intense in T-1 than in T-2 also confirms that there was a difference in its concentration between the ETs. As a result, there was a greater decrease in bicarbonate in T-1, as reported by Kirsch and Sandersen [26], since the changes in bicarbonate and lactate were proportional [25]. Even though this compensatory response occurred together with a drop in pCO_2_, these changes were insufficient to avoid the acidemia observed before the training period. Like the present study, the author also reported a lower lactate production after six [31] and seven weeks [32] of endurance training. However, the acid-base variables did not change when the horses were submitted to low-intensity exercise. This fact demonstrates that the ET used herein was adequate for this type of evaluation, as there was variation along the tests.

Regarding pCO_2_, the absence of change due to training reflects the insufficient adaptation of the respiratory system. No differences were observed in this variable even in racehorses submitted to a training program for 16 weeks [33]. In the current study, it was found that the venous pCO_2_ decreased at the end of the HLac phase and remained low until the end of the exercise in both ETs, indicating the occurrence of hyperventilation. Hypocapnia was also observed in show jumping horses that performed amateur competition [25], as well as in stallions after marcha exercise [34]. Additionally, when evaluating horses after a high-intensity test, it was possible to observe the occurrence of compensatory hyperventilation given the unaltered pCO_2_ [29]. According to some researchers, hyperventilation occurs when the horses stop exercising [24,25], as they are unable to hyperventilate while galloping [35]. However, unlike the present study, in which a reduction in pCO_2_ was reported during the exercise, in these studies, the analyses were performed only before and after the effort. Additionally, our results reveal that, at the end of T-1, the pCO_2_ values were low when compared to T-2. This may be related to the lack of adaptation to the exercise, which led to hyperventilation in order to improve blood oxygenation and thermoregulation, in addition to CO_2_ elimination [25].

After HLac induction, the horses also showed strong ion acidosis associated with SID_4_ reduction, which may have been caused by an increase in anion concentration or a drop in strong cations [20]. This reduction was steeper in T-1, corroborating the fact that the lactate concentration was higher, as there were no electrolyte variations to justify this change. Furthermore, the results indicate that there was no significant loss of fluids. Unlike the ET applied in the present research, in long-term exercise, the variation in SID_4_ occurs mainly as a result of water and electrolyte losses due to sweating. In this case, the hypertonicity of equine sweat can cause an increase in SID_4,_ leading to hypochloremic metabolic alkalosis, which may be masked by a higher lactate concentration [1,36]. However, regardless of the sports modality, horses are subject to mixed imbalances that can be detected using the strong ion approach [25].

In general, the electrolyte changes throughout the exercise were discrete, and with the exception of iCa^2+^, the other electrolytes remained within the physiological limit. The concentrations of Na^+^ and Cl^−^ did not change as a result of the training program, contrary to what was observed in horses that trained for eight weeks, which showed an increase in Na^+^ and a decrease in Cl^−^ [37]. These differences may be attributed to the training period in which the measurement was made. Furthermore, electrolyte changes during exercise can vary depending on the region due to the climatic conditions to which the animals are exposed [38]. Moreover, although there was an increase in Na^+^ in moments of greatest effort intensity, probably because of liquid displacement from the vascular space to the muscles [2], at the end of the ETs, this value returned to baseline.

Despite the effort, the Na^+^ cannot change, as it is essential for the maintenance of the circulating volume, and it is closely regulated by the renin–angiotensin–aldosterone system [39]. Consequently, even in horses that exercised for 30 min at 12 km/h, their concentration did not change 30 min after the end of the exercise [40]. The same happened with animals submitted to a marcha competition for 50 min at an average speed of 9–12 km/h [34]. Herein, the greater stability of this electrolyte in T-2 was possibly due to the activation of neuroendocrine mechanisms of blood volume regulation [41].

With respect to Cl^−^, there was a slight reduction in this electrolyte 20 min after the end of T-1, while in T-2, it decreased at the end of cool-down and returned to baseline. This may have occurred because of the lack of adaptation to the training protocol, with the drop in T-1 possibly being associated with sweating. The decreased Cl^−^ in blood after exercise is commonly observed since this is the electrolyte lost in greater concentration by sweat [42]. Additionally, this reduction may also have been caused by the greater displacement of Cl^−^ to red cells in exchange for HCO_3_^−^, as the latter was reduced at the end of the exercise [43]. Despite this, there was no hypochloremia, as observed in show jumping horses [25], which was in contrast to what was found in racehorses [40], endurance horses after 65 km [36] and stallions after marcha exercise [34]. Even though hydro-electrolyte imbalances can be more serious in long-term exercise, they can also occur in short-term exercise when performed under high-temperature and relatively humid conditions [44].

The baseline concentration of K^+^ decreased as a consequence of training, which was also observed after two weeks of endurance training [41] and hypervolemia. However, during exercise, there was a slight increase in K^+^ in both ETs due to the efflux from the contracting skeletal musculature to the extracellular environment [45]. The elevation of this ion may also be related to the development of fatigue, but only for high-intensity exercises [30]. After the cool-down, K^+^ returned to its baseline value in both ETs, possibly as a result of its return to muscle cells by the action of the Na^+^/K^+^ ATPase pump [46].

As for iCa^2+^, its concentration did not change with training, unlike what was observed by Silva [31] in horses after six weeks of training. However, in the present study, there was an improvement in the animals’ buffering capacity, and no change was found in iCa^2+^. In fact, in both ETs this cation gradually decreased, that is, even though the animals showed higher concentrations of iCa^2+^ at the final moments of the IE phase in T-1, at the end of the exercise, its decrease in relation to its baseline value was equivalent in both ETs (ΔT-1: 0.25 mmol/L; ΔT-2: 0.24 mmol/L). During physical effort, iCa^2+^ displaces towards the interior of the myocytes, leading to muscle contraction caused by actin–myosin interaction [46]. The higher levels of iCa^2+^ observed during T-1 may be attributed to the lack of fitness since the higher production of lactate and the more pronounced metabolic acidosis caused more proteins to be used for the buffering, thus resulting in a greater release of iCa^2+^ [47]. Twenty minutes after the ETs, this ion was still low, but with a tendency to return to its baseline value. A decrease in iCa^2+^ after an effort that was not as long as endurance was also reported in other works [25,34], being attributed to sweat loss [42], or its slow release from the muscles [46].

In the present study, there were no major fluid losses, since both PCV and TPP values remained stable at the end of the exercise and 20 min after. This result does not corroborate that reported for jumping horses, a modality in which the exercise intensity can be compared with that of the ET performed herein [25]. However, the ETs were performed in an air-conditioned room, which may have mitigated the sweat losses. In addition, the horses have large water and electrolyte reserves in their gastrointestinal tract [48], which may have balanced the losses during the effort.

The considerable increase in PCV in both ETs, as well as in TPP and A_tot_ in T-1, occurred in moments of greatest effort, suggesting that these variations can be attributed to the displacement of fluids between compartments [2] and splenocontraction [49], but not to dehydration. Since albumin, globulin and inorganic phosphate were not measured, the A_tot_ was calculated from the TPP. Therefore, as the A_tot_ only considers the contribution of the inorganic phosphate to the TPP concentration, it may not be accurate in cases of abnormal concentrations of other components [26]. The stability of TPP and A_tot_ in T-2 may be explained by the adaptation to the training program, thus reducing the need for protein displacement. The baseline concentration of TPP did not increase with training, unlike what was observed after 45 days [50] and eight weeks [37] of training. It is known that endurance training promotes plasma volume expansion, along with a reduction in PCV [41]. Nonetheless, herein, the PCV did not change, corroborating the result obtained after 45 days of field training [50].

As already mentioned, the literature lacks studies addressing the effect of training on the fluid, electrolyte and acid-base status of horses. As these animals are subject to complex imbalances due to exercise [1,25,34,36,51], the evaluation carried out in the present work, including the traditional and quantitative approaches, helps understand how acid-based homeostasis can be affected by training.

Although the intensity of the ET employed herein was moderate to high, by comparing the values of HCO_3_^−^, BE and pCO_2_ to those obtained in other studies evaluating exercises of same intensity [25,29], it was observed that the imbalances were generally mild to moderate and reversed up to 20 min after the end of ET. In addition, the temperature used was standardized at 38 °C since, in practice, it is not possible to measure body temperature during exercise, even though a temperature rise causes pH reduction [3,51]. Another point to consider is that the horses were submitted to only one type of exercise test and in a temperature-controlled environment, which represent research limitations. Therefore, it would be interesting to evaluate changes under more challenging conditions, with different intensities, durations and climatic conditions. The fact that the animals used in the same analysis were of different sexes is another limiting factor, as evidence points to differences in acid-base balance between sexes [34].

## 5. Conclusions

The results of the present study confirmed that six weeks of endurance training guided by the lactate minimum speed test positively impacted the acid-base status of horses during exercise. Nevertheless, the LMS test protocol employed requires some adaptations in order to greatly improve horses’ fitness in the short term.

## Figures and Tables

**Table 1:** Effort test protocol performed on a high-speed treadmill.

Phase	Moment	Speed (m/s)	Duration	Treadmill Grade (%)
Rest	M0	...	...	...
Warm-up	...	1.4	10 min	0
	...	3.5	5 min	0
HLac	...	10	2 min	10
	M1	1.7	2 min	0
Incremental effort	M2	3	4 min and 27 s	6
	M3	3.5	3 min and 49 s	6
	M4	4	3 min and 20 s	6
	...	4.5	2 min and 58 s	6
	...	5	2 min and 40 s	6
	...	5.5	2 min and 55 s	6
	...	6	2 min and 13 s	6
	...	6.5	2 min and 03 s	6
	...	7	1 min and 54 s	6
	M5	7.5	1 min and 47 s	6
Cool-down	...	3.5	3 min	0
	...	3	2 min	0
	M6	1.7	5 min	0
20 min after	M7	...	...	...

HLac, hyperlactatemia. Moment represents the period of blood sample collection for acid-base status assessment. To analyze the lactate minimum speed method, the blood was collected at a speed from 1.7 m/s to 7.5 m/s.

**Table 2:** Mean ± SD values for the venous blood concentration of lactate, AG and TPP in horses determined during exercise before (T-1) and after (T-2) six weeks of lactate minimum speed-guided training: at rest (M0), at the end of hyperlactatemia (M1), at early stages (M2, M3 and M4) and at the end (M5) of the incremental effort, at the end of cool-down (M6), and 20 min after the end of the test.

Phase	Moment	Lactate (mmol/L)		AG (mmol/L)		TPP (g/dL)	
		T-1	T-2	T-1	T-2	T-1	T-2
At rest	M0	0.91 ± 0.04 ^Ad^	0.93 ± 0.08 ^Ac^	7.38 ± 2.24 ^Ae^	7.29 ± 3.38 ^Ae^	7.37 ± 0.88 ^Ab^	7.22 ± 0.56 ^Aa^
HLac	M1	11.59 ± 5.93 ^Aa^	9.95 ± 3.33 ^Aa^	20.85 ± 5.50 ^Aa^	19.91 ± 5.50 ^Aa^	8.29 ± 1.16 ^Aa^	7.67 ± 0.56 ^Ba^
IE	M2	8.11 ± 6.13 ^Ab^	5.90 ± 3.72 ^Ab^	17.24 ± 5.88 ^Ab^	15.45 ± 5.01 ^Ab^	7.57 ± 0.89 ^Ab^	7.22 ± 0.62 ^Aa^
	M3	4.81 ± 4.52 ^Abc^	2.55 ± 2.49 ^Abc^	13.50 ± 5.18 ^Ac^	11.18 ± 3.12 ^Acd^	7.42 ± 0.81 ^Ab^	7.24 ± 0.73 ^Aa^
	M4	2.88 ± 2.77 ^Acd^	1.35 ± 1.08 ^Ac^	11.86 ± 3.75 ^Acd^	9.78 ± 1.73 ^Acde^	7.44 ± 0.83 ^Ab^	7.22 ± 0.65 ^Aa^
	M5	4.54 ± 1.81 ^Ac^	2.68 ± 0.98 ^Abc^	13.00 ± 2.39 ^Ac^	11.81 ± 2.16 ^Ac^	8.14 ± 0.97 ^Aa^	7.57 ± 0.68 ^Ba^
Cool-down	M6	1.50 ± 0.54 ^Acd^	0.91 ± 0.07 ^Ac^	9.58 ± 2.06 ^Ade^	8.49 ± 1.21 ^Ade^	7.42 ± 0.91 ^Ab^	7.32 ± 0.99 ^Aa^
20 min after	M7	1.49 ± 0.44 ^Acd^	1.56 ± 0.12 ^Ac^	8.20 ± 2.41 ^Ae^	7.91 ± 1.10 ^Ade^	7.57 ± 0.92 ^Ab^	7.34 ± 0.56 ^Aa^

AG, anion gap; TPP, total plasma protein; HLac, hyperlactatemia; IE, incremental effort. Different letters indicate significant differences (*p* < 0.05) between the exercise tests (^A,B^) or times (^a–e^), while shared letters indicate no significant difference.

**Table 3:** Mean ± SD values for venous blood concentration of Na^+^, K^+^, Cl^−^ and iCa^2+^ in horses determined during exercise before (T-1) and after (T-2) six weeks of lactate minimum speed-guided training: at rest (M0), at the end of hyperlactatemia (M1), at early stages (M2, M3 and M4) and the end (M5) of the incremental effort, at the end of cool-down (M6), and 20 min after the end of the test.

Phase	M	Na^+^ (mmol/L)		K^+^ (mmol/L)		Cl^−^ (mmol/L)		iCa^2+^ (mmol/L)	
		T-1	T-2	T-1	T-2	T-1	T-2	T-1	T-2
At rest	M0	135.66 ± 2.91 ^Acd^	136.46 ± 2.03 ^Acd^	3.91 ± 0.29 ^Ade^	3.66 ± 0.24 ^Bb^	105.24 ± 2.50 ^Aa^	104.14 ± 1.12 ^Aabcd^	1.53 ± 0.07 ^Aa^	1.51 ± 0.11 ^Aa^
HLac	M1	139.56 ± 3.89 ^Aa^	139.68 ± 3.39 ^Aa^	4.38 ± 0.18 ^Aab^	4.26 ± 0.18 ^Aa^	104.99 ± 2.14 ^Aab^	103.91 ± 1.14 ^Aabcde^	1.47 ± 0.11 ^Aab^	1.43 ± 0.07 ^Ab^
IE	M2	138.21 ± 3.20 ^Aab^	137.75 ± 2.41 ^Abc^	4.28 ± 0.13 ^Abc^	4.10 ± 0.21 ^Aa^	105.26 ± 2.28 ^Aa^	104.19 ± 1.48 ^Aabc^	1.42 ± 0.07 ^Abc^	1.37 ± 0.06 ^Abcd^
	M3	137.07 ± 2.95 ^Abc^	136.55 ± 1.60 ^Acd^	4.37 ± 0.15 ^Aabc^	4.18 ± 0.19 ^Aa^	105.16 ± 1.92 ^Aa^	104.44 ± 0.95 ^Aa^	1.41 ± 0.07 ^Abc^	1.35 ± 0.04 ^Abcd^
	M4	136.95 ± 2.59 ^Abc^	136.54 ± 0.90 ^Acd^	4.47 ± 0.09 ^Aab^	4.18 ± 0.19 ^Ba^	105.15 ± 1.75 ^Aa^	104.76 ± 1.44 ^Aa^	1.42 ± 0.06 ^Abc^	1.34 ± 0.05 ^Bcdef^
	M5	137.82 ± 2.50 ^Aab^	137.76 ± 1.26 ^Abc^	4.69 ± 0.19 ^Aa^	4.42 ± 0.21 ^Ba^	104.28 ± 2.13 ^Aabc^	103.81 ± 1.21 ^Aabcde^	1.36 ± 0.06 ^Acd^	1.28 ± 0.07 ^Bdef^
Cool-down	M6	135.38 ± 2.32 ^Acd^	135.41 ± 0.46 ^Ad^	3.54 ± 0.28 ^Adef^	3.39 ± 0.18 ^Ab^	104.69 ± 1.85 ^Aabc^	102.65 ± 0.76 ^Aef^	1.28 ± 0.11 ^Ae^	1.27 ± 0.06 ^Af^
20 min after	M7	135.78 ± 1.98 ^Acd^	135.95 ± 0.87 ^Acd^	3.26 ± 0.84 ^Afg^	3.22 ± 0.25 ^Ab^	103.56 ± 2.35 ^Acd^	102.84 ± 0.74 ^Acde^	1.39 ± 0.08 ^Ac^	1.34 ± 0.07 ^Adef^

HLac, hyperlactatemia; IE, incremental effort; M, moment. Different letters indicate significant differences (*p* < 0.05) between the exercise tests (^A,B^) or times (^a–g^), while shared letters indicate no significant difference.

## Data Availability

The data that support the findings of this study are available upon request from the corresponding author.

## Supplementary Materials

The following supporting information can be downloaded at: https://www.mdpi.com/article/10.3390/ani13203290/s1, Table S1: Formulae used; Figure S1: Bland–Altmann plots; Table S2: Reference values in the venous blood of horse. References [20,21,52] are cited in the supplementary materials.
