# Supplementary material for: Effect of Lactate Minimum Speed-Guided Training on the Fluid, Electrolyte and Acid-Base Status of Horses
Source: Animals (Basel). 2023 Oct 21;13(20):3290. doi: 10.3390/ani13203290 (PMC10603943; doi:10.3390/ani13203290)
Supplement: Supplementary file 1 [file animals-13-03290-s001.zip › Table S1.pdf]

Table S1. Formulae used.

Variable	Formulae
SID_4 [20]	$SID_4 = (Na^+ + K^+) - (Cl^- + lactate)$
AG [21]	$AG = (Na^+ + K^+) - (Cl^- + HCO_3^-)$
SIG [21]	$SIG = \left(\frac{[A_{tot}]}{[1 + 10^{6.65-pH}]}\right) - AG$
A_{tot} [20]	$A_{tot} = 2.24 \times PPT\ (g/dL)$

20. Constable, P.D. A simplified strong ion model for acid-base equilibria: application to horse plasma. *J. Appl. Physiol*. **1997**, 83, 297–311.

21. Constable, P.D.; Hinchcliff, K.W.; Muir, W.W. Comparision of anion gap and strong ion gap as predictors of unmeasured strong ion concentrion in plasma and serum from horses. *Am. J. Vet. Res*. **1998**, 59, 881–887.
