# Supplementary material for: Effect of Lactate Minimum Speed-Guided Training on the Fluid, Electrolyte and Acid-Base Status of Horses
Source: Animals (Basel). 2023 Oct 21;13(20):3290. doi: 10.3390/ani13203290 (PMC10603943; doi:10.3390/ani13203290)
Supplement: Supplementary file 1 [file animals-13-03290-s001.zip › Table S2.pdf]

Table S2. Reference values in venous blood of horses.

Variable	Reference range
pH	7.32-7.46 [52]
pCO_2 (mmHg)	38-46 [52]
HCO_3^- (mmol/L)	24-30 [52]
BE (mmol/L)	0-6.0 [52]
Lactate (mmol/L)	<2 [52]
Na^+ (mmol/L)	132-146 [52]
K^+ (mmol/L)	3.0-5.0 [52]
Cl^- (mmol/L)	99-109 [52]
iCa^{2+} (mmol/L)	1.4-1.7 [52]
PPT (g/dL)	6.0-7.7 [52]
SID_4 (mmol/L)	±40 [20]
AG (mmol/L)	8-13 [21]
SIG (mmol/L)	-2 to +6 [21]
A_{tot} (mmol/L)	12-15 [20]

References

52. Constable, P.D.; Hinchcliff, K.W.; Done, S.H.; Grünberg, W. (2017) *Veterinary medicine: a textbook of the disease of cattle, horses, sheep, pigs, and goats*, 11th ed., Elsevier Ltd, St. Louis.

20. Constable, P.D. A simplified strong ion model for acid-base equilibria: application to horse plasma. *J. Appl. Physiol.* **1997**, 83, 297–311.

21. Constable, P.D.; Hinchcliff, K.W.; Muir, W.W. Comparision of anion gap and strong ion gap as predictors of unmeasured strong ion concentrion in plasma and serum from horses. *Am. J. Vet. Res.* **1998,** 59, 881–887.
