# Supplementary figures and images for: Effect of Lactate Minimum Speed-Guided Training on the Fluid, Electrolyte and Acid-Base Status of Horses
Source: Animals (Basel). 2023 Oct 21;13(20):3290. doi: 10.3390/ani13203290 (PMC10603943; doi:10.3390/ani13203290)

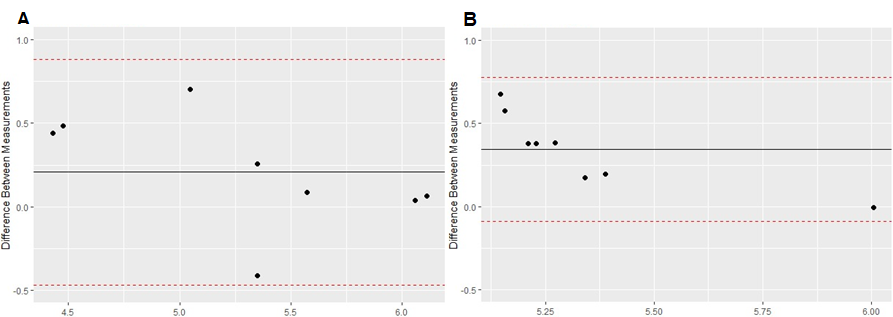

Supplement: Supplementary file 1 [file animals-13-03290-s001.zip › Figure S1.tif]
